# New Mobilities and Psychology: Why are We Still Not on the Move?

**DOI:** 10.5964/ejop.v16i2.3117

**Published:** 2020-05-29

**Authors:** Vlad P. Glăveanu

**Affiliations:** a Webster University Geneva, Geneva, Switzerland; b Bergen University, Bergen, Norway

**Keywords:** mobility, mobilities, psychology, self, identity, learning, imagination, creativity

## Abstract

The new mobilities paradigm has been influential within the social sciences for the past two decades. And yet, psychology is undoubtably slow to incorporate mobility as a key lens through which to consider its subject area. In this editorial, I will make the case that we would benefit greatly from focusing more on personal, collective and psychological mobilities and the kinds of conceptual, methodological and practical challenges they raise. To illustrate this, I briefly discuss the notions of self and identity, learning, and imagination and creativity. Final conclusions are offered regarding a late but welcomed ‘mobilities turn’ in psychological science.

The coronavirus pandemic and the lockdowns imposed, at the moment of writing, on half the world population, raise important questions for psychologists. These range from knowing the factors that facilitate and hinder social distancing to the mental health and well-being implications of home confinement and social isolation (Lima et al., 2020; Sritharan & Sritharan, 2020; Swami & Barron, 2020). How do people cope with this new and unexpected situation and with the fear of getting infected or spreading the virus? How do we deal, more generally, with the massive transformation of our private and professional lives? What is *immobility* doing to us, as individuals and as societies?

The question mobility has never been more pressing. As our physical mobility is seriously restricted, other forms of ‘movement’ flourish. People find ways to move within the house in unexpected ways, including running marathons on balconies^i^ or climbing the Everest on staircases^ii^. More importantly, we keep in touch with others through a variety of communication, virtual and digital technologies and enjoy the experience of being in other places and situations through movies, games, and online discussions. Above all, we engage in psychological forms of mobility, helped by our memory, imagination and creativity, and participate in a series of activities and scenarios we are being denied access to at the moment. Are all of these forms of mobility?

If we start from the basic assumption that movement describes a change of position from A to B, or a trajectory, then we should unpack the notion of *position* first (see also Martin, 2006). Typically, we imagine these as physical, represented by where our bodies are. But, as social and cultural beings, we also occupy, at once, other types of positions in the world. We adopt different social roles and move between them in any given situation, and we also hold symbolic positions, enabled by our imagination, in the here-and-now, the not-yet-here, the elsewhere and even the nowhere (Jovchelovitch, Priego-Hernandez, & Glăveanu, 2013). Each one of these facilitate specific relations to oneself, others, and the world, often conceptualised as *perspectives*. Most of all, we are never fixed within a certain position but, in the course of action and interaction, exchange and move between them, sometimes occupying more than one position and holding more than one perspective at a time (see, e.g., Position Exchange Theory; Gillespie & Martin, 2014).

Mobilities, then, need to be much more widely defined than physical movement and the paradigmatic case of migration. In psychology, we certainly have a well development literature focused on the situation of migrants and refugees (Ryan, Dooley, & Benson, 2008; Zittoun, Levitan, & Cangiá, 2018) and a branch concerned more widely with transport and transportation (Gärling, Gärling, & Loukopoulos, 2002; Van Acker, Van Wee, & Witlox, 2010). But we are lacking an understanding of how mobility cuts across *all* our areas of concern as researchers of inherently *mobile* minds, individual, groups, and societies^iii^. In other words, psychologists by and large are late to engage with what became known in the social sciences as the ‘new mobilities’ paradigm. Spearheaded by sociologist and geographers, this approach “examines the diverse mobilities of peoples, objects, images, information and wastes; and of the complex interdependencies between, and social consequences of, these diverse mobilities” (Urry, 2000, p. 1).

From the broad definition before we can conclude that at least the mobility of people, images and information should directly interest psychologists. Yet, in fact, the scope for cross-fertilisation is much wider, considering a more detailed description of new mobilities themes:

- “the *corporeal* travel of people for work, leisure, family life, pleasure, migration and escape, organised in terms of contrasting time-space patterns ranging from daily commuting to once-in-a-lifetime exile;
- the physical movement of *objects* include food and water to producers, consumers and retailers; as well as the sending and receiving of presents and souvenirs;
- the *imaginative* travel effected through the images of places and peoples appearing on and moving across multiple print and visual media and which then construct and reconstruct visions of place, travel and consumption;
- *virtual* travel often in real time transcending geographical and social distance and forming and reforming multiple communities at-a-distance;
- *communicative* travel through person-to-person messages via personal messages, postcards, texts, letters, telegraph, telephone, fax and mobile” (Urry, 2011, pp. 4-5).

All of the above are potential themes of research for psychologists, from migration and different forms of communication to the use of virtual spaces, the exchange of objects and the processes of imagination. What a mobilities focus would require of the discipline, though, is not only to see its themes of research in movement, but as *constituted by movement*. And this is where psychology in particular, with its largely static, abstract and universalistic focus^iv^, has difficulties adopting ontologies based on states of flux, transformation, and the role of context. Sociologists used this opportunity to rethink society from a static, bounded entity to a dynamic network of flows (see Sheller & Urry, 2006). Geographers saw a chance to update their already existing geographies of movement and transport geographies (Cresswell, 2011). Anthropologists started seriously investigating the relationship between imagination and migration (Salazar, 2010) and archaeologists the one between migration and cultural change (Heitz & Stapfer, 2017).

In psychology it is primarily *sociocultural researchers* who began, in recent years, to discuss the importance of movement and mobility (e.g., see Wagoner, Chaudhary, & Hviid, 2015). This is not surprising given that cultural or sociocultural psychology starts from the interdependence between mind and culture and considers this interdependence in a developmental manner (Valsiner, 2007). In other words, the focus falls on the temporal trajectory of people’s interactions with others and their construction and use of material and symbolic tools (this is why, e.g., a life-course approach is preferred; see Zittoun, Valsiner, Gonçalves, Salgado, Vedeler, & Ferring, 2013). Recently, Gillespie and Zittoun (2013) offered a mobility-based approach to how we integrate and diversity experiences. They start from the pragmatist premise that, as we physically move through our environment, we accumulate experiences that we can later on use as resources for our imagination. As such, physical and psychological mobilities are intrinsically linked. However, in their framework, the two can also be relatively independent from each other given that, for instance, we often ‘travel’ mentally to other people, places and events while remaining relatively immobile in the here and now. Their sharp separation between proximal and distal experiences is questionable though, especially if we consider the fact that our experience integrates *both* and imagination remains, at all times, an embodied process (see also Glăveanu, Karwowski, Jankowska, & de Saint-Laurent, 2017).

In building a mobilities-based psychology, it is important to take into account the temporality of human action and its different ‘levels’. In a very recent book (see Glăveanu, 2020), I propose a study of mobility and possibility focused on four inter-related *temporal dimensions*: the phylogenetic roots of migrating people, the sociogenetic dynamic of ideas and innovations on the move, the ontogenetic aspect of personal mobilities, and the microgenetic processes of psychological movement. In each instance, I considered how mobility begets possibility or, to put it differently, how movement opens up new horizons for our thinking of and action in the world. This is not to say, of course, that there is a linear, unidirectional causality between mobility and explorations of the possible (through imagination, creativity, and innovation); also, that there are no instances in which acts of mobility constrict our sense of possibility. But the ontological premise remains – without moving between or exchanging positions, no new perspectives can come about.

Whether we are talking about human experience or possibility, working within the new mobilities paradigm doesn’t mean just adding a new point of focus – on movement – to a long list of existing psychological research topics. An integration of mobility into our theories and methods transforms how we define, study, and cultivate *each and every* one of them. I will briefly illustrate this with the help of three themes – self and identity, learning, creativity and imagination – as an invitation to reflect on the constitutive role of movement for our social and psychological life.

The *self* has long been an area of research for psychologists, typically conceived as a distinguishing feature of human beings due to its reflective nature. The self emerges, according to Mead (1934), when we become able to see ourselves as another person would; in other words, when we can adopt the perspective of others upon the self (see also Gillespie, 2006a). What we are presented with here is a basic case of social and psychological mobility: repositioning ourselves or relocating to the position of the other. This movement seems abstract and psychological, but it is, in fact, rooted in highly embodied forms of activity, for example in the play and games we engage in during early childhood (Gillespie, 2006b). Equally, *identities* are often conceived of as relatively stable and dependent on categorization and group membership. What research over the past decades consistently shows is that identities are much more mobile, flexible and contextual than we initially thought (see Reicher, 2004). And the latter can be accounted for in terms of movements, social, psychological and oftentimes physical, between groups and categories.

*Learning* and education more broadly would also benefit immensely from the mobilities approach. While theories of learning have recognised for a long time their contextual or situated nature (e.g., Arnseth, 2008), further steps can be taken towards understanding the learner, his or her context, and the processes of learning in ‘movement’. Some useful insights have been developed over the past years in the direction of theorising and empirically studying learning trajectories and learning networks (see Leander, Phillips, & Taylor, 2010). These encourage us to consider not only what and how students learn, but how their learning and interests have their own history and movement across time and space. They also come to challenge old ideas in education that view the classroom as a container and as the privileged place of learning.

Finally, I made reference several times before to *imagination* and *creativity*. These possibility expanding phenomena don’t take place, as many wrongly assume, ‘inside the head’ of the person who imagines or goes through moments of creative insight. They are, in fact, premised on all sorts of embodied, social, psychological and cultural mobilities. Zittoun and Gillespie (2015) captured this through the loop model of the imagination that basically conceives it as an act of looping in and out of immediate experience and towards the past, the future, and alternative worlds. These can be interpreted as different positions and the loop as a fundamental unit of psychological movement. In creativity research, I theorised the creative process as a dialogue of perspectives (see Glăveanu, 2015) premised on re-positioning. These ‘micro-mobilities’ of creators, often invisible to outside observers, build on, continue and contribute to personal ‘mezzo-mobilities’ (e.g., travel, visits) and societal ‘macro-mobilities’ (e.g., social change).

In the end, why should psychologists pay more attention to new mobilities research and try to develop mobility-based accounts of their own topics? As I tried to argue here, the latter would radically *transform* the way we consider minds, bodies, and societies and how we recognise their richness, complexity, and ontological basis in movement. This would challenge static, universalistic theories on the one hand, and open up a new vocabulary for thinking about the psychic on the other, including trajectories and transitions, re-positioning and position exchange, flows, scapes, and networks. Most of all, it would inspire a methodological renaissance that has been highly influential in the social sciences and led, until now, to the development of a range of new methods such as mobile ethnographies, time–space diaries and the investigation of virtual spaces (see Sheller & Urry, 2006). Mobilities scholars can also benefit from engaging more with psychological theories and methods, especially when it comes to building a deeper understanding of personal and psychological forms of mobility. In the end, even if the time to start moving, in psychology, was decades ago, it’s never too late to join fellow sociologists, geographers and anthropologists in their journey through a young paradigm and contribute fully to its growth and future trajectory.

## References

[r1] Arnseth H. C. (2008). Activity theory and situated learning theory: Contrasting views of educational practice. Pedagogy, Culture & Society, 16(3), 289–302. 10.1080/14681360802346663

[r2] Cresswell T. (2011). Mobilities I: Catching up. Progress in Human Geography, 35(4), 550–558. 10.1177/0309132510383348

[r3] Fischer P. A. Malmberg G. (2001). Settled people don’t move: On life course and (im‐) mobility in Sweden. International Journal of Population Geography, 7(5), 357–371. 10.1002/ijpg.230

[r4] Gärling T. Gärling A. Loukopoulos P. (2002). Forecasting psychological consequences of car use reduction: A challenge to an environmental psychology of transportation. Applied Psychology, 51(1), 90–106. 10.1111/1464-0597.00080

[r5] Gillespie, A. (2006a). *Becoming other: From social interaction to self-reflection*. Greenwich, CT, USA: Information Age Publishing.

[r6] Gillespie A. (2006 b). Games and the development of perspective taking. Human Development, 49(2), 87–92. 10.1159/000091334

[r7] Gillespie A. Martin J. (2014). Position exchange theory: A socio-material basis for discursive and psychological positioning. New Ideas in Psychology, 32, 73–79. 10.1016/j.newideapsych.2013.05.001

[r8] Gillespie A. Zittoun T. (2013). Meaning making in motion: Bodies and minds moving through institutional and semiotic structures. Culture and Psychology, 19(4), 518–532. 10.1177/1354067X13500325

[r9] Glăveanu V. P. (2015). Creativity as a sociocultural act. The Journal of Creative Behavior, 49(3), 165–180. 10.1002/jocb.94

[r10] Glăveanu, V. P. (2020). *Mobilities and human possibility*. London, United Kingdom: Palgrave.

[r11] Glăveanu, V. P., Karwowski, M., Jankowska, D. M., & de Saint-Laurent, C. (2017). Creative imagination. In T. Zittoun & V. P. Glăveanu (Eds.), *The Handbook of imagination and culture* (pp. 61-86). New York, NY, USA: Oxford University Press.

[r12] Heitz, C., & Stapfer, R. B. (Eds.). (2017). *Mobility and pottery production: Archaeological and anthropological perspectives*. Leiden, Netherlands: Sidestone Press.

[r13] Jovchelovitch S. Priego-Hernandez J. Glăveanu V. P. (2013). Constructing public worlds: Culture and socio-economic context in the development of children’s representations of the public sphere. Culture and Psychology, 19(3), 323–347. 10.1177/1354067X13489320

[r14] Leander K. M. Phillips N. C. Taylor K. H. (2010). The changing social spaces of learning: Mapping new mobilities. Review of Research in Education, 34(1), 329–394. 10.3102/0091732X09358129

[r15] Lima C. K. T. de Medeiros Carvalho P. M. Lima I. D. A. S. de Oliveira Nunes J. V. A. Saraiva J. S. de Souza R. I. Neto M. L. R. (2020). The emotional impact of Coronavirus 2019-nCoV (new Coronavirus disease). Psychiatry Research, 287, 112915. 10.1016/j.psychres.2020.112915 32199182 PMC7195292

[r16] Lubkemann S. C. (2008). Involuntary immobility: On a theoretical invisibility in forced migration studies. Journal of Refugee Studies, 21(4), 454–475. 10.1093/jrs/fen043

[r17] Martin J. (2006). Positions, perspectives, and persons. Human Development, 49(2), 93–95. 10.1159/000091335

[r18] Mead, G. H. (1934). *Mind, self and society*. Chicago, IL, USA: University of Chicago Press.

[r19] Reicher S. (2004). The context of social identity: Domination, resistance, and change. Political Psychology, 25(6), 921–945. 10.1111/j.1467-9221.2004.00403.x

[r20] Ryan D. Dooley B. Benson C. (2008). Theoretical perspectives on post-migration adaptation and psychological well-being among refugees: Towards a resource-based model. Journal of Refugee Studies, 21(1), 1–18. 10.1093/jrs/fem047

[r21] Salazar N. B. (2010). Towards an anthropology of cultural mobilities. Crossings: Journal of Migration & Culture, 1(1), 53–68.

[r22] Sheller M. Urry J. (2006). The new mobilities paradigm. Environment & Planning A, 38(2), 207–226. 10.1068/a37268

[r23] Sritharan J. Sritharan A. (2020). Emerging mental health issues from the novel Coronavirus (COVID-19) pandemic. Journal of Health and Medical Sciences, 3(2), 157-162. Retrieved from https://ssrn.com/abstract=3577545. 10.31014/aior.1994.03.02.109

[r24] Swami, V., & Barron, D. (2020). *Analytic thinking, rejection of coronavirus (COVID-19) conspiracy theories, and compliance with mandated social-distancing: Direct and indirect relationships in a nationally representative sample of adults in the United Kingdom*. Retrieved from https://osf.io/nmx9w

[r25] Urry, J. (2000). *Sociology beyond societies: Mobilities for the twenty-first century*. London, United Kingdom: Routledge.

[r26] Urry, J. (2011). Does mobility have a future? In M. Grieco & J. Urry (Eds.), *Mobilities: New perspectives on transport and society* (pp. 3–20). Surrey, United Kingdom: Ashgate.

[r27] Valsiner, J. (2007). *Culture in minds and societies: Foundations of cultural psychology*. New Delhi, India: Sage.

[r28] Van Acker V. Van Wee B. Witlox F. (2010). When transport geography meets social psychology: Toward a conceptual model of travel behaviour. Transport Reviews, 30(2), 219–240. 10.1080/01441640902943453

[r29] Wagoner, B., Chaudhary, N., & Hviid, P. (2015). Cultural psychology on the move. In B. Wagoner, N. Chaudhary, & P. Hviid (Eds.), *Integrating experiences: Body and mind moving between contexts* (pp. ix-xv). Charlotte, NC, USA: Information Age Publishers.

[r30] Zittoun, T., & Gillespie, A. (2015). *Imagination in human and cultural development*. London, United Kingdom: Routledge.

[r31] Zittoun T. Levitan D. Cangiá F. (2018). A sociocultural approach to mobile families: A case study. Peace and Conflict: Journal of Peace Psychology, 24(4), 424–432. 10.1037/pac0000313

[r32] Zittoun, T., Valsiner, J., Gonçalves, M. M., Salgado, J., Vedeler, D., & Ferring, D. (2013). *Human development in the life course: Melodies of living*. Cambridge, United Kingdom: Cambridge University Press.

